# Polymorphous Adenocarcinoma of the Parotid Gland: A Rare Entity in Asians With a Unique Cystic Presentation

**DOI:** 10.1002/ccr3.70441

**Published:** 2025-04-21

**Authors:** Ibadat Preet Kaur, Devendra Pankaj, Meena Santosh, Jitendra Sharan, Neha Singh, Anand Marya

**Affiliations:** ^1^ Department of Dentistry ESIC Medical College and Hospital Alwar Rajasthan India; ^2^ Department of Otorhinolaryngology ESIC Medical College and Hospital Alwar Rajasthan India; ^3^ Department of Pathology ESIC Medical College and Hospital Alwar Rajasthan India; ^4^ Department of Dentistry All India Institute of Medical Sciences Bhubaneswar Odisha India; ^5^ Department of Otorhinolaryngology RDJM Medical College and Hospital Muzzafarpur Bihar India; ^6^ Faculty of Dentistry University of Puthisastra Phnom Penh Cambodia

**Keywords:** cytopathology, diagnostic dilemma, parotid tumors, polymorphous adenocarcinoma, polymorphous low‐grade adenocarcinoma, salivary gland tumor

## Abstract

Polymorphous adenocarcinoma (PAC) of the parotid is a rare entity with minimal reported incidences in the Asian population. It generally presents as a solid tumor and is considered a diagnostic dilemma due to morphological diversity and variable histological patterns. This article reports a unique fluid‐filled PAC of the parotid gland mimicking a cystic lesion in an Indian male, with a review of relevant literature. A 64‐year‐old male patient presented with a slow‐growing swelling of the left parotid region for the last 10 years. The cytology report suggested a cystic swelling. The patient underwent superficial parotidectomy. A detailed histopathological examination confirmed it as a classical variant of PAC. He remains disease‐free over 24 months post‐surgery follow‐up.

## Graphical abstract

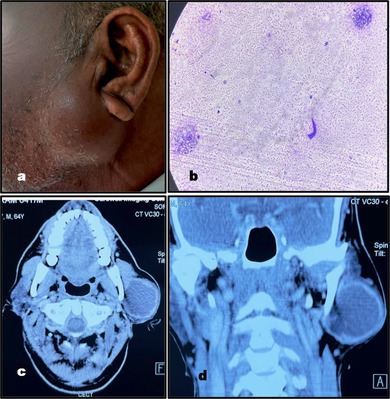

**Summary**

- PAC of parotid gland is a rare malignancy with racial predilection and can present as a cystic swelling.
- Cytological diagnosis for diffuse cystic lesions has minimal sensitivity due to hypocellular smears of disparate entities with similar findings.
- A non‐specific, non‐diagnostic, cytomorphologically benign lesion can actually be a malignant tumor of any grade.

## 1 Introduction

Polymorphous adenocarcinoma is a rare malignant epithelial tumor of salivary glands that was first recognized as a distinct clinicopathological entity, “Lobular carcinoma”, by Freedman and Lumerman in 1983 [1]. Evans and Batsakis suggested the term polymorphous low‐grade adenocarcinoma (PLGA) for the tumor in 1984 [2]. Due to its polymorphous histology and indolent clinical behavior, the term was also adopted by WHO in its second classification of histological typing of salivary gland tumors in 1991 [3]. The fourth update of WHO classification (2017) of salivary gland tumors has removed the specific (Low) grade from the name, imparting the flexibility in pathological grading and allowing for the recognition of a broader spectrum within an entity [4, 5]. It also categorized the indolent PLGA as classical variant of polymorphous adenocarcinoma (PAC), being defined as “a malignant epithelial tumor characterized by cytological uniformity, morphological diversity, an infiltrative growth pattern, and low metastatic potential.” [5] The term PAC has been retained in most recent, fifth update(2022) for the clinically, histologically and molecularly heterogeneous group of tumors of salivary glands; and hence will be further used in the present case report [6].

It is reported in the third–seventh decade of life, with over 90% of cases occurring above 40 years with a mean age of 61.3 years at diagnosis [7]. The tumor has a clear female predilection in a ratio of 2.15:1 [8]. Although rare, it is the second most common malignancy of the minor salivary glands (MiSG) after mucoepidermoid carcinoma; located frequently in the posterior hard and soft palate (60% of cases, range: 49%–87%). The incidences of occurrence in labial and buccal mucosa, retromolar trigone, tongue, floor of mouth, nasal cavity, paranasal sinuses, larynx, trachea, and bronchi have also been reported [7, 8, 9]. It may occasionally originate in major salivary glands, particularly the parotid, in 3% (range: 0%–9%) of cases [7, 9]. The tumor also shows racial preponderance with 75% cases reported in Caucasians and less than 2% (0.5%–1.6%) in Asians [8, 10]. As per the author's literary search, total of 44 case reports of parotid PAC (Table 1) have been published till 01/06/2023. These include nine cases by Asian authors (Case no. 1–5, 7, 8, 10, 11) and only three cases (Case No. 1, 3, 5) from India till date. The present article describes the rare occurrence of PAC presenting with a unique cystic appearance in the parotid gland of an Indian male. A comprehensive review of the limited relevant literature has also been included in it.

**TABLE 1:** Summary of cases of PLGA of parotid gland reported in the literature.

S. no.	Case no.	Age/sex	Origin	Pre‐operative diagnosis	Treatment	Follow‐up	Outcome	Metastasis	Author/year/country
1	1	16/M	Ex‐PMA	PMA on FNAC	Surgery, RT	33 months	NAD	ND	Khosla D. et al. (2017) [10] India
2	2	21/F	De novo	Low‐grade papillary neoplasm on FNAC	Surgery	NM	NM	NM	Shreshtha et al. (2012) [11] Nepal
3	3	25/F	De novo	Epithelial Parotid tumor on Punch biopsy	Surgery, RT	1 year	NAD	No	Krishnamurthy et al. (2011) [12] India
4	4	35/M	De novo	Multicystic tumoral mass on USG	Surgery	NM	NM	NM	Gelincik I et al. (2010) [13] Turkey
5	5	25/M	De novo	Chronic Parotitis	Surgery	NM	NM	NM	Arathi N (2009) [14] India
6	6	60/M	De novo	PMA on FNAC	Surgery, RT	48 months	NAD	No	Godoy R et al. (2007) [15] Mexico
7	7	52/F	De novo	Parotid Mass on CT	Surgery, RT	50 months	NAD	No	Uemaetomari I (2007) [16] Japan
	8 (Two interventions)	Ist‐55/F	Ex‐PMA	Parotid tumor on CT and MRI	Surgery	6 years	Local recurrence	NM	
		IInd‐61/F		NM	Surgery, RT	33 months	NAD	No	
8	9	45/M	NM	PMA on trans‐oral biopsy	Radical Surgery, RT	5 years	NAD	No	O'Rourke et al. (2006) [17] New England
9	10	79/M	De novo	ACC on excisional biopsy	Surgery	14 months	NAD	No	Nagao et al. (2004) [18] Japan
	11 (Two interventions)	Ist‐65/M	De novo	Carcinoma ex‐PMA	Surgery	46 months	Local recurrence	NM	
		IInd—NM		NM	Surgery, RT	4 months	NAD	NM	
10	12	65/F	De novo	NM	NM	NM	NM	NM	Tamiolakis D et al. (2004) [19] Greece
11	13	65/F	NM	ACC on FNAC	NM	NM	NM	NM	Gibbons et al. (1999) [20] Texas
	14	66/M		PLGA on FNAC					
12	15	43/M	De novo	Parotid tumor on CT scan	Surgery, RT	20 months	NAD	No	Barak P A et al.(1998) [21] New England
13	16	69/F	De novo	NM	Surgery, RT	2 years	NAD	No	Puxeddu et al. (1998) [22] Italy
15	17 (Two interventions)	Ist‐54/F	De novo	NM	Surgery, RT	Lost to follow‐up Returned after 11 years.	Local recurrence	No	Merchant et al. (1996) [23] UK
		IInd‐NM			Surgery	4 years	NAD	No	
14	18–39 (22 cases)	37–83 (58.8) M:F‐1:7	De novo	NM	NM	1.5–12 years (mean‐5.2 years) 13 cases available for follow‐up	Local recurrence in 4 cases	To regional lymph nodes in one case	Kemp et al. (1995) [24] USA
15	40	85/F	De novo	Carcinoma ex‐PMA	Surgery	72 months	Local reccurrence	No	Ritland F et al. (1993) [25] USA
16	41	70F	Ex‐PMA	—	Surgery	9 months	NAD	No	Mark J et al. (1992) [26] Sweden
17	42	68/M	Ex‐PMA	NM	Surgery, RT	18 months	NAD	No	George MK et al. (1991) [27] UK
18	43	50/F	de novo	NM	Surgery, RT	30 months	NAD	No	Miliauskas JR et al. (1991) [28] South Australia
19	44	69/M	Ex‐PMA	FNAC inconclusive	Surgery	12 months	NAD	No	Mark J et al. (1991) [29] Sweden

Abbreviations: ACC, adeniod cystic carcinoma; Ex‐PMA, ex‐pleomorphic adenoma; FNAC, fine needle aspiration biopsy; NAD, no abnormality detected.; NM, not mentioned; PMA, pleomorphic adenoma; RT, radiotherapy.

## 2 Case History/Examination

A 64‐year healthy, smoker male patient presented with the chief complaint of left cheek swelling since the last 10 years. History revealed that it started as a small painless nodule that progressed slowly over time without any associated bleeding, ulceration, or associated symptoms of dysphasia, dysphonia, dysphagia, otalgia, odontalgia, or odynophagia. He visited several regional professionals for the same and had been prescribed antibiotics for the aural and dental infections. The drainage through a local incision had also been attempted 3 years back without any significant results. No record of previous intervention was available with him.

**FIGURE 1:**
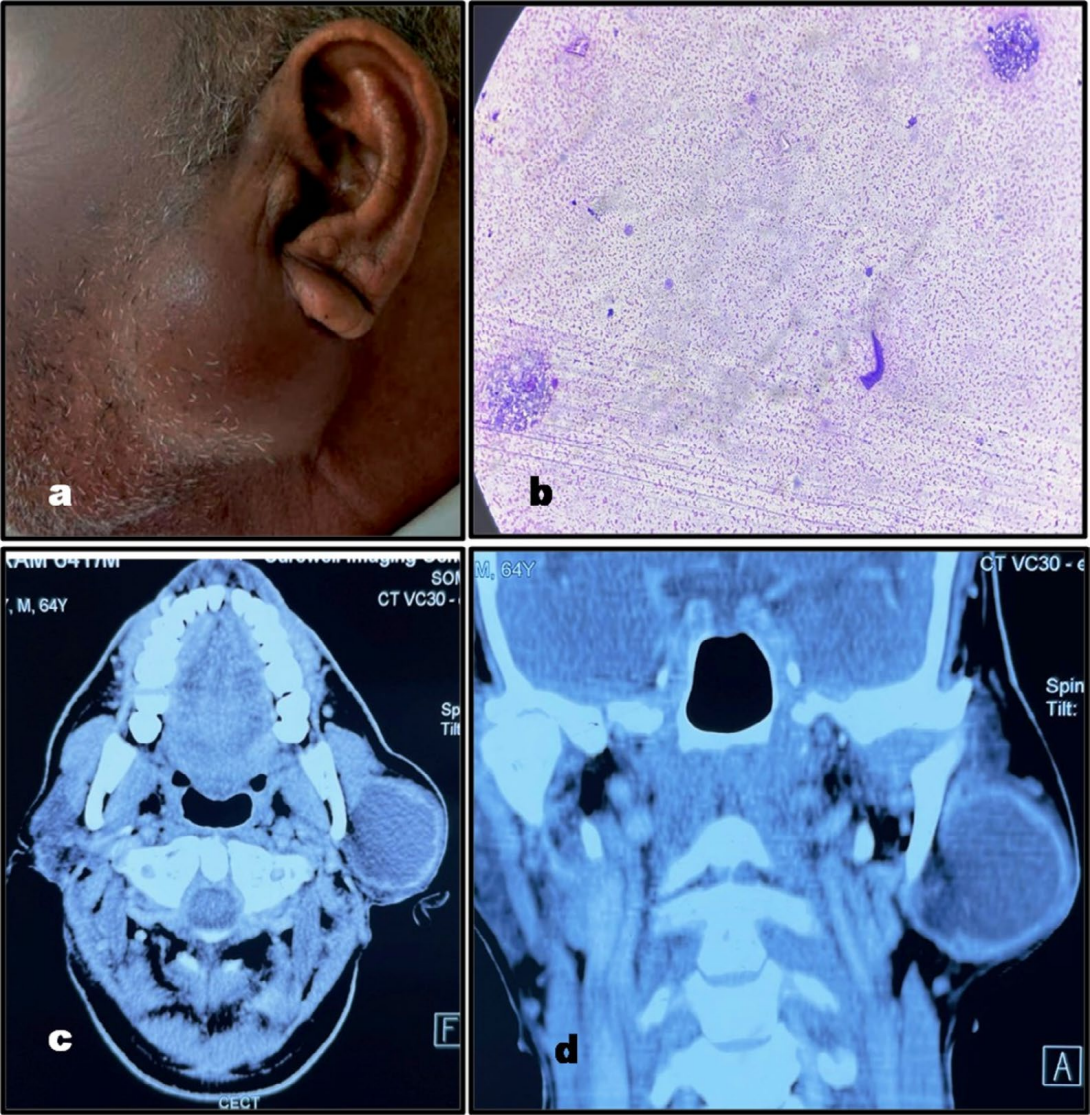
Pre‐operative presentation of the pathological swelling: (a) Clinical view. (b) cytological smear‐cystic fluid with scanty macrophages (100×). (c) CT scan axial view—showing left parotid swelling. (d) CT scan coronal view showing left parotid swelling.

Clinical examination recorded a swelling of 4 × 4 cm size in the left preauricular region with extension to the infra‐auricular area (Figure 1a). It was non‐tender, non‐fluctuant, localized, firm in consistency, and had well‐defined margins without any fixation to the underlying structures on palpation. There was absence of cervical lymphadenopathy and the functions of the facial nerve were maintained, without any evidence of weakness. No other significant etiological factor was identified on the detailed head and neck examination. The hematological and biochemical liver and renal function tests were within normal range. The viral markers were negative for HIV and Hepatitis B infection. Fine needle aspiration was done using 26‐gauge needle and 8 mL of brown fluid was aspirated. A rapid filling of the swelling immediately after FNAC was also reported by the pathologist. The cytological smears were prepared from centrifuged fluid and were Giemsa stained. They were hypocellular with scanty macrophages scattered in the serous fluid. The smears were categorized “Non‐Diagnostic” [30] due to the absence of any characteristic cells or patterns, and a cytological diagnosis of benign cystic lesion in broad sense has been established (Figure 1b). Pre‐operative contrast‐enhanced computed tomography (CECT) recorded a well‐defined cystic mass of 43 × 41 × 39 mm dimension in the left parotid with peripheral enhancement and extension of the deep lobe (Figure 1c,d) A provisional diagnosis of benign lymphoepithelial cyst or Warthin's tumor was suggested.

## 3 Methods

**FIGURE 2:**
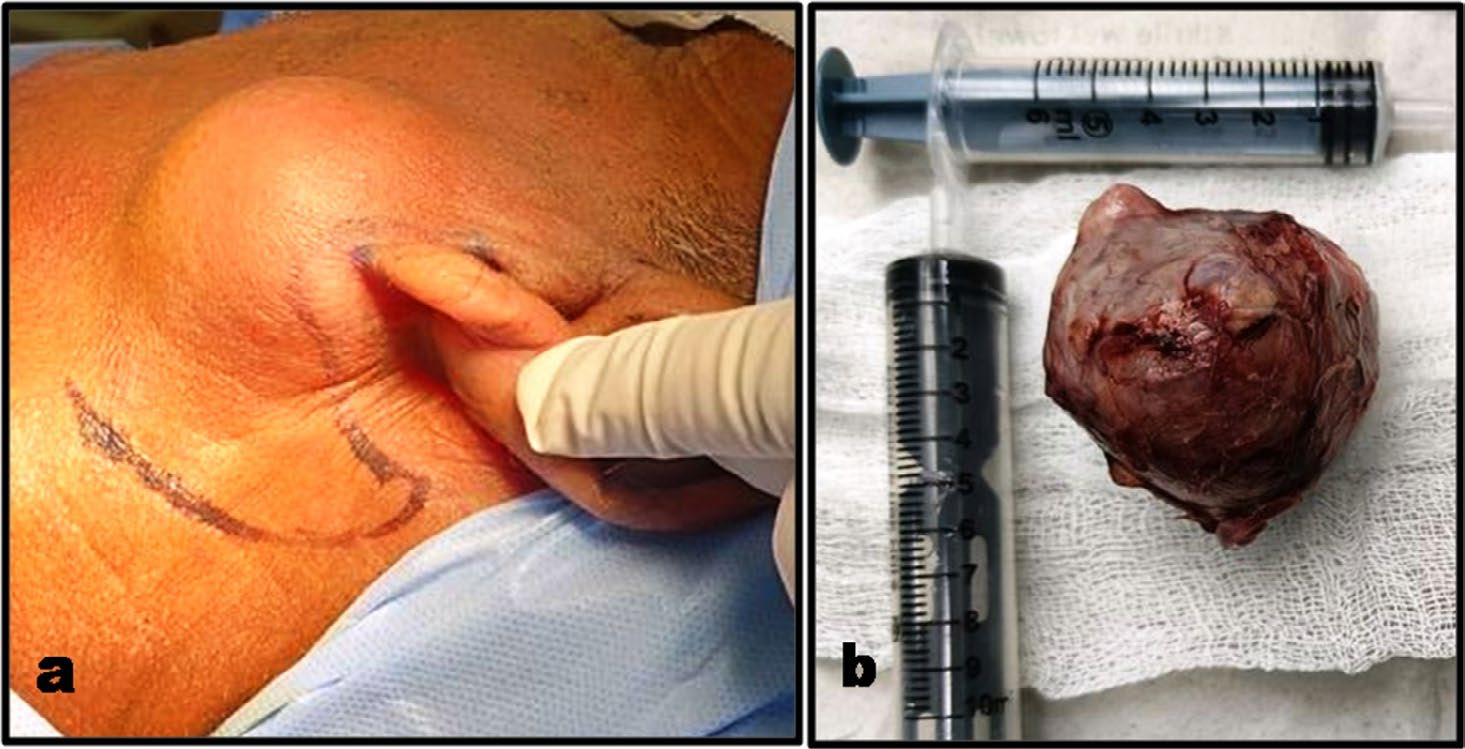
Intra‐operative presentation: (a) Modified Blair incision. (b) Soft tissue pathological mass of size 5 × 6 cm.

Given the abovementioned findings, the case was considered to be a benign lesion and written informed consent for superficial parotidectomy under general anesthesia was obtained. The site was approached through a modified Blair incision (Figure 2a). The musculoaponeurotic flap was elevated anteriorly. The facial nerve was identified at the tragal pointer and preserved. A single, 5 × 6 cm soft, cystic mass was excised in toto and sent for histopathology (Figure 2b). Suturing was done after achieving hemostasis.

**FIGURE 3:**
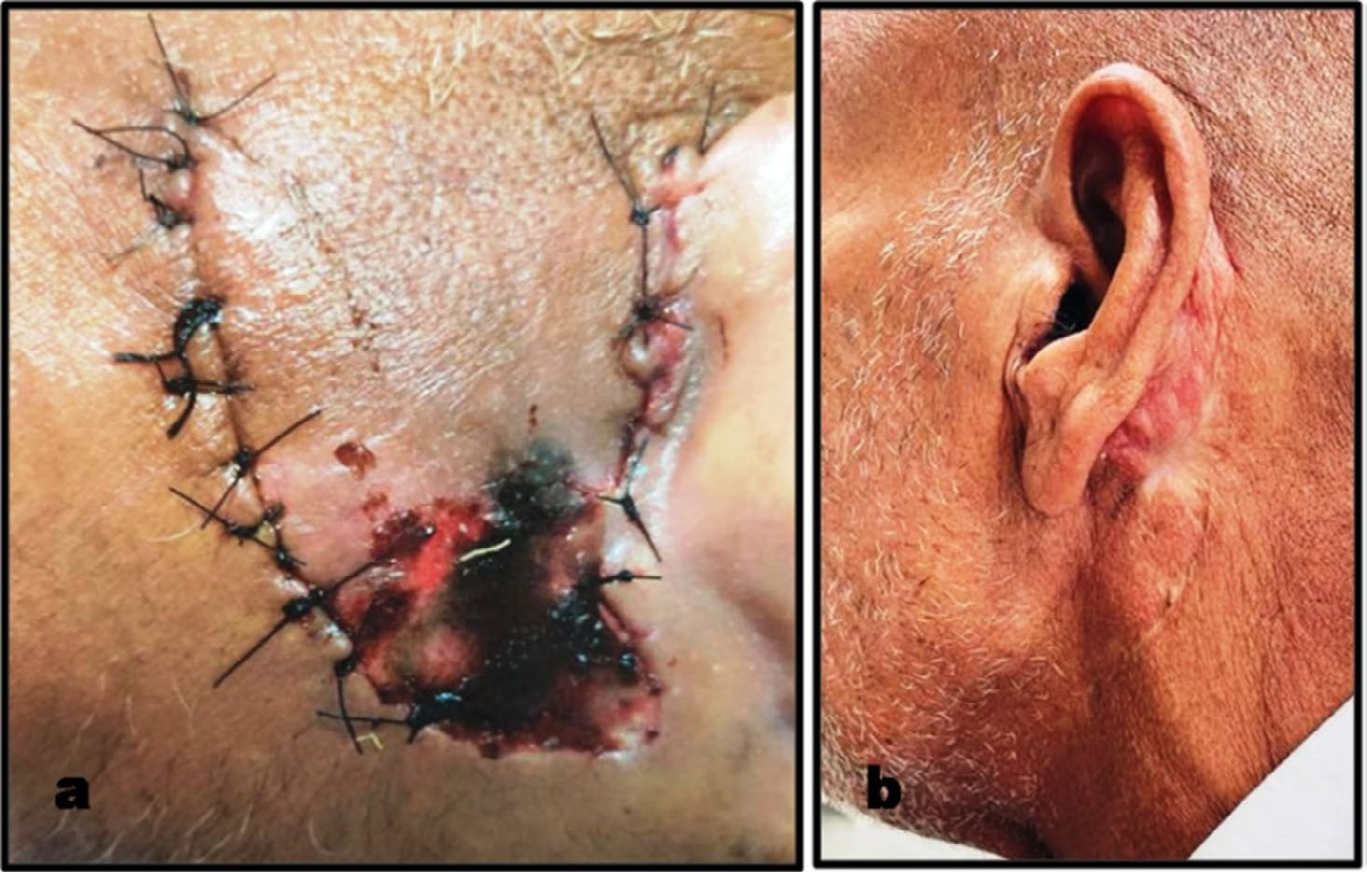
Postoperative presentation of the surgical site: (a) Flap necrosis on the fifth day postoperative. (b) Residual scar at the time of discharge.

Post‐operative healing was uneventful without any major surgical complications. Flap necrosis was noted on day 5 (Figure 3a), which was successfully managed conservatively. Facial nerve function was gradedI as per the House‐Brackmann scale. Any segmental weakness, changes in facial soft tissues, and synkinesis were non‐existent. Suture removal was done on the 10th day, and the patient was discharged on the 25th day (Figure 3b).

**FIGURE 4:**
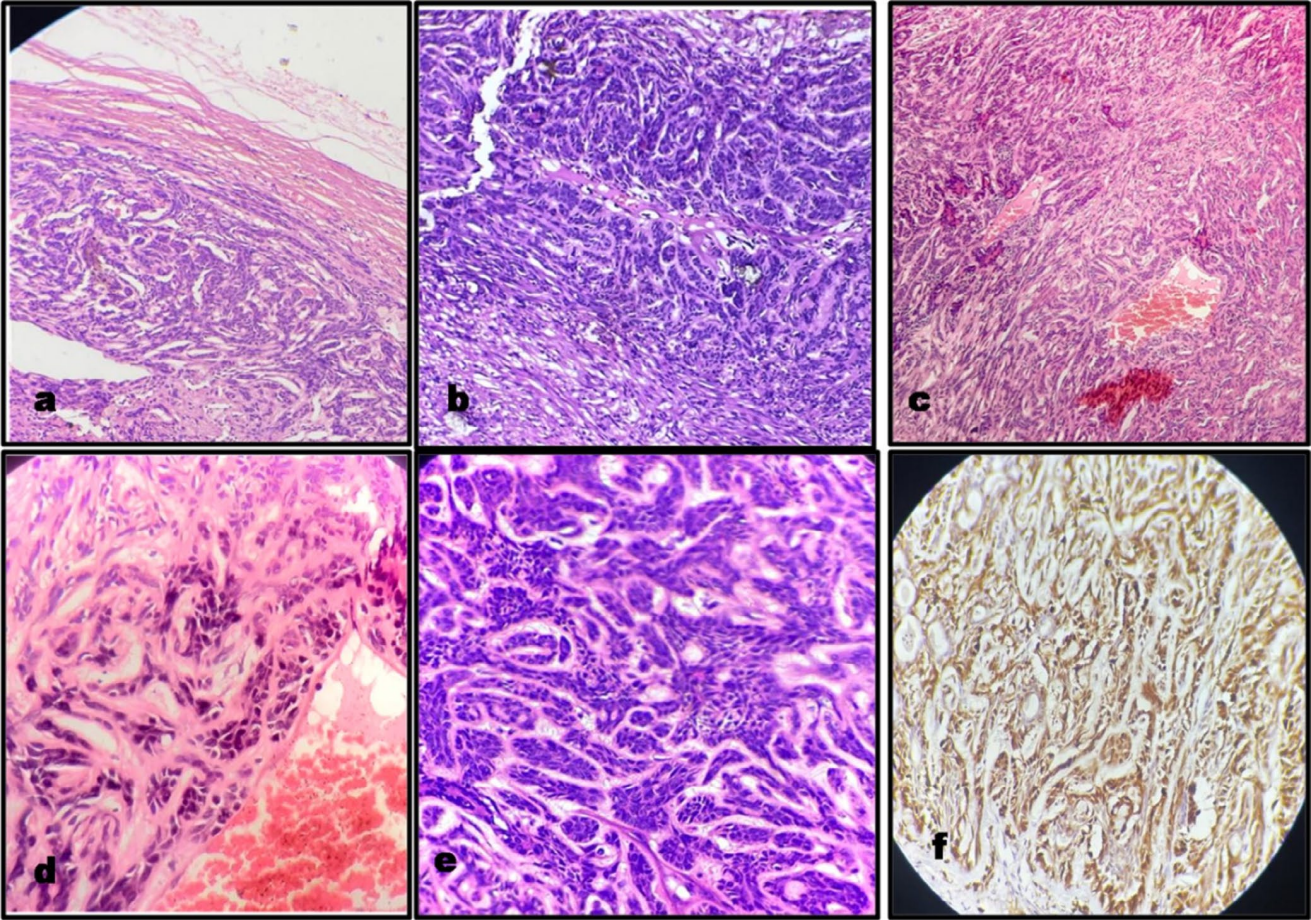
Histopathological images of biopsy: (a) Peripheral infiltration of tumor—H&E stain; 100×. (b and c) Arrangement of the tumor cells in various architectural patterns—H&E stain; 100×. (d and e) Round tumor cells with indistinct borders, eosinophilic cytoplasm, round to oval nuclei, and inconspicuous nuclei—H&E stain; 100×. (f) Tumor cell positive with IHC stain—S100 antigen, 400×.

The histopathology reported that a 5 × 4 × 3 cm mass exhibited a hemorrhagic, fluid‐filled cut section macroscopically. Microscopically, an infiltrative pattern at the periphery was evident (Figure 4a). The cells were arranged in a variety of architectural patterns including tubular, trabecular, solid, and cribriform (Figure 4b,c). The small to medium‐sized round tumor cells had a uniform shape, an indistinct border, and eosinophilic cytoplasm (Figure 4d,e). Their nuclei were round to ovoid and contained open vesicular nuclear chromatin and inconspicuous nucleoli (Figure 4d,e). Considering the histological features, the final diagnosis of PAC was made. It was further confirmed by the positivity of S‐100 on immunohistochemistry (Figure 4f). The patient was informed about the diagnosis and its potential complications and was referred to a higher center for post‐operative radiotherapy. However, anticipating complications, radiotherapy, or any other further management was rigidly refused by him. His post‐operative CT recorded the signs of chronic parotitis without any evidence of residual disease. The patient was recalled monthly for the first 3 months, followed by every 3 months at the present institute. He is completely asymptomatic with the absence of any evidence of recurrence, metastasis, or associated complications at the final follow‐up of 24 months. The scheduled follow‐ups are expected to be carried out in the future also to assess the long‐term outcome of the treatment.

## 4 Discussion

Parotid gland swellings are considerably taken as one of the most difficult areas of diagnostic pathology due to variable clinical manifestations and heterogeneous and overlapping patho‐morphological images [4]. PAC, classical variant, characteristically presents as an oval, firm to solid, un‐encapsulated, circumscribed slow‐growing swelling with variable histological patterns and minimal nodal metastasis [7, 8]. The origin has been described as de novo or as an ex pleomorphic adenoma (ex‐PMA), from the terminal (intercalated) duct cells of salivary glands [8]. FNAC is recommended to be the first‐line tissue‐based testing procedure for establishing the pre‐surgical diagnosis for major salivary gland PAC [7, 20]. The procedure is challenging because of the presence of limited tissue access and variable cyto‐architectural findings [20]. The smears are hypercellular with branched papillary clusters and sheets of uniform cells with moderately eosinophilic cytoplasm; round‐to‐oval nuclei with bland or absent nucleoli; finely stippled chromatin; and abundant hyaline globules within the matrix [20]. These findings were completely absent and FNAC was “non‐diagnostic” in our case. Due to the absence of any specific radiographic features, the role of imaging is also limited to the assessment of its origin, local extent, and any regional or distant metastasis rather than distinguishing a distinct diagnosis. PLGAs of the parotid gland have mostly been provisionally misinterpreted in the literature (Table 1), evidently revealing the lack of specificity of the pre‐operative diagnostic testing for them. The present case was also provisionally diagnosed as a cystic lesion of the parotid gland.

The different lesions exhibiting the cystic appearance in the parotid gland have been categorized by WHO (2017) as non‐neoplastic cysts, benign tumors with macrocystic formation, and malignant tumors with macrocystic change [31]. The cystic presentation of PAC in the present case has not been included in the categorization and hence can be considered a distinct appearance of a rare entity [31]. Salivary duct cyst, the most common non‐neoplastic cyst, is a well‐defined, thin‐walled cavity filled with histocyte‐laden mucinous fluid [31, 32]. Lymphoepithelial cysts can be unilateral or HIV‐associated, bilateral lesions having foamy histocytes and polymorphous lymphoid cells in a serous/proteinaceous background [31, 32]. Macrocystic Warthin's tumor has a well‐defined thin capsule enclosing a variable proportion of solid and cystic components. The cases of cystic transformation in the entire tumor have also been reported in the literature [33]. FNAC demonstrates lymphocytic, non‐mucinous fluid and characteristic 2‐D sheets or papillary‐like clusters of oncocytic cells lining the ductal structures [32]. Pleomorphic adenoma is slow‐growing tumor with well‐demarcated smooth or lobulated contour. The focal to entire cystic transformation secondary to tumor degeneration is preferably recorded in large adenomas. Cytologically, epithelial and myoepithelial cells in the myxochondroid stroma are evident [32].

Considering the indolent clinical course, intact facial nerve functions, radiologically well‐defined cystic swelling without hard tissue erosion, absence of any local or distant metastatic spread and non‐diagnostic cytology negative for atypical, oncocytic, epithelial, and myoepithelial cells; the present swelling was provisionally interpreted as a lymphoepithelial cyst. Predicting a benign neoplasm with negligible metastatic potential, it was surgically managed by a minimally invasive option of superficial parotidectomy with preservation of the facial nerve. The pathology was disparate from the pre‐operative diagnosis and reported as the rare polymorphous low‐grade adenocarcinoma of the parotid gland. It was graded as T3N0M0 and concluded to be the classical variant as per WHO 2017 classification due to clinical evidence of slow growth with the absence of local or distant metastasis over the last 10 years and the presence of small to medium cells in multiple patterns, targetoid cells, and < 30% of cribriform areas [6]. The diffuse positivity for the S‐100 stain, which is reported to be associated with more than 90% of reported cases, further confirmed the diagnosis [34].

The papillary variant of Acinic cell carcinoma (ACC‐PCV) is also a rare, low‐grade, malignant macrocystic tumor of the parotid gland with poor long‐term prognosis [31]. It has a predilection for young females (16–40 years) and presents as a painless, slow‐growing swelling with well‐defined margins [35]. The cytological smears are variegated with papillary epithelial proliferations and multicellular papillary clusters [32, 35]. Additionally, one or more types of hobnail acinar, intercalated, vacuolated, nonspecific glandular, and clear cells can be seen in variable proportions histopathologically [31]. Another salivary malignancy presenting as well‐defined mass with a major cystic component is low‐grade mucoepidermoid carcinoma [31]. Histologically, multiple epithelium‐lined cystic spaces with lymphoid proliferation, papillary infoldings with mucous cells, and scant intermediate cells are observed [36]. Both of these malignancies are invariably negative for S‐100. Epithelial myoepithelial carcinoma is a low‐grade S‐100 positive salivary gland malignancy presenting with cystic changes in 30% of cases [31, 37]. The double‐layered arrangement of inner ductal and outer myoepithelial cells is evident microscopically. Macrocystic carcinoma ex‐pleomorphic adenoma emerges as a sudden rapid increase in the growth rate of long‐standing parotid swelling with neurological symptoms and microscopic calcification and hyalinization [31, 32]. Four isolated incidences of carcinomatous transformation of parotid salivary duct cyst (SDC) that is, undifferentiated [38], mucoepidermoid [39], myoepithelial, [37] and adenocarcinoma [40] ex–SDC have also been reported in the literature. The morphologically similar cystic lesions of the parotid gland must be distinguished from one another due to different management protocols. Compared to solid tumors, cytological diagnosis reportedly has low sensitivity and yields higher false negative results due to hypocellularity and overlapping cytomorphological findings [32]. Cytological heterogeneity additionally intricately affects the initial assessment in the present case. Characteristic histopathological polymorphous patterns of cell arrangement complemented by S‐100 antibody positivity established the differential diagnosis from the abovementioned malignancies.

The PAC has been stratified as a low‐grade malignancy amongst salivary gland carcinomas [41]. Recommended treatment, independent of its location, consists of the wide local surgical excision, with additional neck dissection only in cases with cervical lymphadenopathy [42]. As the initial treatment plan was framed, diagnosing it as a benign lesion, non‐accomplishment of the wide surgical excision without the consideration for surgical margins is the main limitation of the present case. Adjunctive radiotherapy, performed for such tumors in the literature was also denied by the patient. The role of postoperative radiotherapy is ambiguous in the treatment of PAC, and it may diminish the local recurrence of tumors having high‐risk features, closed margins, perineural invasion, and so forth [8, 16]. Moreover, the recurrence may take years to develop in PACs (Table 1), having the absence of adverse features with negative or even positive margins [8, 42, 43]. As the tumor was a classical variant suspected to have an indolent course, and the postoperative CT scan also recorded minimal residual disease, the patient is kept under the regular 3‐month follow‐up to detect the earliest sign of any anticipated complications in the future.

## 5 Conclusion

Macrocystic salivary gland tumors are diagnostic challenges to the pathologist and management conundrums to surgeons and oncologists. PAC of the parotid gland is a rare entity in the Asian population and can manifest as a macrocystic variant. The available pre‐operative investigations have a limited role in its diagnosis, and a detailed histopathological examination is the standard for confirmation.

## Author Contributions

**Ibadat Preet Kaur:** conceptualization, data curation, formal analysis, supervision, validation, visualization, writing – original draft, writing – review and editing. **Devendra Pankaj:** data curation, funding acquisition, investigation, methodology, project administration, supervision, validation, visualization, writing – original draft, writing – review and editing. **Meena Santosh:** conceptualization, data curation, formal analysis, funding acquisition, supervision, validation, visualization, writing – original draft, writing – review and editing. **Jitendra Sharan:** funding acquisition, investigation, methodology, project administration, validation, visualization, writing – original draft, writing – review and editing. **Neha Singh:** conceptualization, data curation, resources, software, supervision, validation, visualization, writing – original draft, writing – review and editing. **Anand Marya:** conceptualization, data curation, formal analysis, validation, visualization, writing – original draft, writing – review and editing.

## Disclosure

Guarantor: All the authors are nominated guarantors of the manuscript.

## Ethics Statement

Ethical approval was not required for this study.

## Consent

Written informed consent was obtained from the patient to publish this report in accordance with the journal's patient consent policy.

## Conflicts of Interest

The authors declare no conflicts of interest.

## Data Availability

Data related to the manuscript can be provided by the corresponding author on reasonable request.
